# Development of a short food frequency questionnaire to assess diet quality in UK adolescents using the National Diet and Nutrition Survey

**DOI:** 10.1186/s12937-020-00658-1

**Published:** 2021-01-12

**Authors:** Sarah Shaw, Sarah Crozier, Sofia Strömmer, Hazel Inskip, Mary Barker, Christina Vogel, the EACH-B Study Team

**Affiliations:** 1 MRC Lifecourse Epidemiology Unit, Southampton General Hospital, University of Southampton, Tremona Road, Southampton, SO16 6YD UK; 2 NIHR Southampton Biomedical Research Centre, University of Southampton and University Hospital Southampton NHS Foundation Trust, Southampton, UK

**Keywords:** Adolescents, Dietary assessment, Diet quality, National Diet and nutrition survey, Short food frequency questionnaire

## Abstract

**Background:**

UK adolescents consume fewer fruits and vegetables and more free sugars than any other age group. Established techniques to understand diet quality can be difficult to use with adolescents because of high participant burden. This study aimed to identify key foods that indicate variation in diet quality in UK adolescents for inclusion in a short food frequency questionnaire (FFQ) and to investigate the associations between adolescent diet quality, nutritional biomarkers and socio-demographic factors.

**Methods:**

Dietary, demographic and biomarker data from waves 1–8 of the National Diet and Nutrition Survey rolling programme were used (*n*=2587; aged 11–18 years; 50% boys; *n*=≤997 biomarker data). Principal component analysis (PCA) was applied to 139 food groups to identify the key patterns within the data. Two diet quality scores, a 139-group and 20-group, were calculated using the PCA coefficients for each food group and multiplying by their standardised reported frequency of consumption and then summing across foods. The foods with the 10 strongest positive and 10 strongest negative coefficients from the PCA results were used for the 20-group score. Scores were standardised to have a zero mean and standard deviation of one.

**Results:**

The first PCA component explained 3.0% of variance in the dietary data and described a dietary pattern broadly aligned with UK dietary recommendations. A correlation of 0.87 was observed between the 139-group and 20-group scores. Bland-Altman mean difference was 0.00 and 95% limits of agreement were − 0.98 to 0.98 SDs. Correlations, in the expected direction, were seen between each nutritional biomarker and both scores; results attenuated slightly for the 20-group score compared to the 139-group score. Better diet quality was observed among girls, non-white populations and in those from higher socio-economic backgrounds for both scores.

**Conclusions:**

The diet quality score based on 20 food groups showed reasonable agreement with the 139-group score. Both scores were correlated with nutritional biomarkers. A short 20-item FFQ can provide a meaningful and easy-to-implement tool to assess diet quality in large scale observational and intervention studies with adolescents.

**Supplementary Information:**

The online version contains supplementary material available at 10.1186/s12937-020-00658-1.

## Background

Most adolescents consume high quantities of energy-dense, nutrient-poor foods and have been shown to have less healthy dietary behaviours than other age groups [1, 2]. Only 8% of UK adolescents aged 11–18 years are reaching the ‘5 A DAY’ fruit and vegetable recommendations, and their intake of free sugars make up on average 14% of total energy intake, almost three times the recommended level [2]. Poor health behaviours during adolescence, including having an unhealthy diet, not only have the potential to negatively impact the individual’s immediate and future health status, but also the health of their future offspring [3]. Dietary intake is a multidimensional and complex behaviour. The assessment of dietary patterns embraces the interrelationships and synergies between foods and has been shown to be more strongly associated with health outcomes, than consideration of a single nutrient or food in isolation [4, 5].

Assessing dietary intake in any age group can be challenging and many established dietary assessment techniques can be difficult to implement because of high participant and time burdens [6]. Self-report data are considered to be useful and appropriate for dietary analysis when energy and individual nutrient intake are not the main focus of the research, such as with diet patterns analysis [7]. Diet diaries and 24-h recalls are often considered the preferred self-report data collection tool providing they have been validated against objective measures of nutritional status [7]. The requirement to provide detailed dietary records as an accurate representation of an individual’s diet, ideally over a number of days, means that these methods can ask a great deal of the participant [8]. In addition, these methods are likely to be completed more comprehensively by individuals from more advantaged backgrounds [7]. When conducting research with adolescents, additional barriers such as low levels of motivation, varying levels of cognitive development and lack of willingness to cooperate can all be potential obstacles to accurate data collection [8]. Few tools exist to assess diet quality in adolescents from varying socio-economic backgrounds in large-scale population studies. Food Frequency Questionnaires (FFQs) can, however, be used to address a number of these issues since they require only a single-time point assessment which is designed to capture habitual diet and foods irregularly consumed [6].

Two approaches have been used for dietary patterns analysis: i) a priori and ii) a posteriori. A priori approaches compare dietary intake to an existing dietary framework, such as food-based dietary guidelines [9]. Many a priori defined dietary indices have been developed for adults and may therefore not be suitable for use with adolescents [10]. One a priori diet quality index, however, has specifically been created for adolescents using data from Europe, to assess level of adherence to the Flemish Healthy Eating Guidelines [11]. Application of this index involves collection of dietary data via 24 -h recalls or diet diaries. As a result, this diet quality index may prove difficult to implement in large-scale observational and intervention studies, which are often limited by resource, time and financial restraints. A priori patterns are typically assigned a grade based on how well they meet recommended dietary guidelines. This approach may potentially miss details about dietary variation and important information about intake of energy-dense, nutrient-poor foods. A posteriori approaches, on the other hand, use statistical methods to create data-driven patterns based on actual dietary intakes [9, 12]. One statistical approach commonly used is principal component analysis (PCA). PCA has been shown to be an effective method for developing short food frequency questionnaires as it allows for the identification of key foods, both healthy and unhealthy, that contribute most strongly to a given dietary pattern. It has been applied to data from age groups including young women, older adults and young children [13–15].

The study reported in this paper used data from a nationally representative dietary dataset for UK adolescents aged 11–18 years to address four research aims:

1. To identify a dietary pattern that describes diet quality in UK adolescents;
2. To create a reduced-item diet quality score (for potential use as a short food frequency questionnaire for assessing diet quality in UK adolescents);
3. To investigate the associations between the diet quality score and objective biomarkers of nutritional status;
4. To investigate the associations between the diet quality score and adolescent socio-demographic characteristics.

## Methods

The reporting of this study follows the STROBE-nut framework [16]. The STROBE-nut checklist can be found in Additional file 1.

### National Diet and Nutrition Survey

The National Diet and Nutrition Survey (NDNS) rolling programme is a repeated cross-sectional survey of food consumption and nutrient intake of the general UK population that has been running since 2008. Data from years 1–8 (2008–2016) of the NDNS rolling programme were used for this study. These data were accessed through the UK Data Service archives [17].

The NDNS aims to collect nationally representative data annually from roughly 500 adults and 500 children, aged 1.5 years and older. All residential addresses in the UK are clustered into small geographical areas based on postcode. A list of households is randomly selected from each geographical area. In households with more than one eligible participant, an adult and a child are randomly selected to participate. At some addresses, only children were selected to take part in an attempt to achieve equal numbers of adults and children [18].

In the first stage of the survey, participants were visited by an interviewer to complete a face-to-face questionnaire that covers lifestyle and socio-demographic characteristics of both the household and individual. Participants were also asked to keep a four consecutive day estimated (unweighed) diet diary. In the diet diary, participants were asked to record all food and drinks consumed both at home and away from home along with estimated portion size, brand names or ingredients for homemade meals. Individuals aged 12 years and older were asked to complete the diary themselves, while parents/carers were asked to keep the diary for younger participants. Participants received two follow-up visits from the interviewer to address any questions from the participants and deal with possible omissions and missing data. Data were collected throughout the year to account for potential seasonal variations. The diet diaries were coded by trained coders from the NDNS research team in order to classify the reported food and drink items into 155 food groups that are reported in the NDNS food-level dataset. Participants who successfully completed stage one of the survey, which required at least three completed diet diary days, were also given the opportunity to participate in stage two. This stage involved a home visit from a study nurse to collect a blood and urine sample for biomarker analyses and took place within 2–4 months of finishing stage one of data collection. Fasting blood samples were collected by venepuncture for eligible and willing participants by a trained nurse. Participants who were not willing to fast or who had diabetes were asked to provide a non-fasting blood sample. Participants in the years 1–5 of the NDNS rolling programme were asked to collect all urine passed in a 24-h period. Collections were classified as complete, either by P-amino-benzoic acid (PABA) or by participant claim. The 24-h urine samples were not collected for participants in Years 6–8 of the rolling programme due to a change in the NDNS survey protocol.

### Food frequency questionnaire development

The NDNS food-level dataset for adolescents aged 11–18 years, which included 155 food groups, was used for the analysis to address the first research aim. Vitamins, minerals and artificial sweeteners were removed from the dataset (16 variables) as this study aimed to only assess the consumption of food and drink products. There were 139 food group variables used in this analysis. The frequency of consumption of each of these 139 food groups was calculated for each participant. A small number of participants (2%) completed three, rather than four, days of diet diaries. The consumption frequencies for these participants were multiplied by 1.33.

In order to address the first research aim, PCA was used to identify the dietary pattern describing the main axis of variation within the data. PCA is a data reduction method that produces new variables that are linear combinations of the original dietary variables and maximises the explained variance within the data [19]. PCA was performed on the weekly consumption frequencies of the 139 food groups for all adolescent participants. The first component of the PCA, the main axis of variation in the dietary data, was used to create individualised 139-group diet quality scores by multiplying the PCA coefficients for all the food groups by each individual’s standardised frequency of consumption and summing across all 139 food groups.

In order to address the second research aim, 20 food groups were selected comprising those with the 10 strongest positive and 10 strongest negative coefficients in the PCA performed on the 139 food groups. The 10 strongest positive and 10 strongest negative were chosen to enable the score to detect reductions in consumption of less healthy foods, as well as increases in consumption of healthier foods. A 20-group diet quality score was then calculated by multiplying the coefficients (from the 139-food group PCA) for these 20 food groups by each individual’s standardised frequency of consumption and summing across the 20 food groups. A 20 food group FFQ has been shown to be a pragmatic and acceptable length of questionnaire to assess diet quality in young women and children [13, 14]. Additional file 2 provides an explanation of the steps involved in calculating the diet quality score.

In order to ensure the 20 NDNS food groups were suitable to use in a self-administered FFQ, cognitive think-aloud interviews were conducted with eight adolescents, aged 13–14 years, who were students at a state secondary school based in Southampton, UK. Cognitive interviews provide a method of ensuring that survey questions are interpreted by participants in a consistent manner and in the way intended by the researcher [20]. During the think-aloud interviews, participants were asked to complete the questionnaire and verbalise the process by which they decided their response to each item.

### Nutritional blood biomarkers

In order to address the third research aim, NDNS blood biomarkers from the NDNS individual-level dataset were used; 37% of adolescent participants provided a blood sample and 42% provided a urine sample.

25-Hydroxy Vitamin D was measured by liquid chromatography–mass spectrometry (LC-MS/MS) analysis using the Diasorin Liaison methods. Vitamin C fluorescence was measured on the BMG Labtech FLUOstar OPTIMA plate reader. Individual carotenoids were determined by high performance liquid chromatography using methods derived from Sowell et al. [21]. Serum folate was measured by LC-MS/MS. Vitamin B12 measures were performed using the ADVIA Centaur B12 assay. The holoTC assay manufactured by AXIS Shield was used to generate holotranscobalamin measurements. The homocysteine assays were performed using the Siemens BN ProSpec® system. Urinary sodium and potassium were measured using ion-specific electrodes on the Siemens Dimension® Xpand clinical chemistry system with the QuikLYTE® module. Further details on the laboratory and assay methods used have been published as part of the NDNS report [22, 23].

A priori, a total of fifteen biomarkers was selected for inclusion in this study based on published literature. 25-hydroxy vitamin D [11, 24], total carotenoids (lutein, alpha-cryptoxanthin, beta-cryptoxanthin, lycopene, beta-carotene, alpha-carotene) [11, 25, 26] and total serum folate [13] were selected because they showed moderate to strong associations with healthy dietary patterns in previous literature. Holotranscobalamin [11], vitamin B12 [27], homocysteine [27, 28] and vitamin C [11, 15, 29] were also included based on moderate, yet sometimes inconsistent, associations observed in previous literature. Weak associations between the diet quality score and urinary sodium and potassium, were expected [30].

### Socio-demographic variables

In order to address the final research aim, socio-demographic variables from the NDNS data set were used. Detailed background information, such as age and ethnicity, was collected during the face-to-face nurse administered interview. Ethnicity was categorised into white and non-white. Information about household income was collected from the main food provider for the household. An equivalised household income variable was included in the NDNS dataset which adjusts household income to account for different resource demands such as household size and composition. Equivalised household income was described in relation to a £27,000 cut-point. This cut point was selected as it is just above the median UK household income in 2012, the midpoint for the data in this study [31]. Index of Multiple Deprivation (IMD) scores were calculated for each household based on the home address. IMD is the official measure of relative deprivation for small areas in England and combines information from seven domains including i) income deprivation; ii) employment deprivation; iii) education, skills and training deprivation, iv) health deprivation and disability; v) crime; vi) barriers to housing and services; vii) living environment deprivation [32]. The NDNS dataset divided IMD scores into quintiles and used to provide an estimated deprivation level for each household.

### Statistical analysis

In order to create dietary scores with a normal distribution, the same transformations were applied to both the 139-group and 20-group scores: after adding five to each variable, they were logged, and then standardised by subtracting the mean and dividing by the standard deviation. The scores therefore have a mean of zero and a standard deviation of one. Normally distributed continuous variables were summarised using means and standard deviations, and categorical variables were summarised using frequencies and percentages. The association between the full 139-group diet quality score and the reduced 20-group diet quality score was calculated using Pearson’s correlation coefficient. Bland-Altman 95% limits of agreement [33] were also calculated to assess the level of agreement between the 139-group and 20-group diet quality score. Spearman’s correlations were used to assess the relationships between the diet quality scores and biomarkers. Mean (SD) 20-group diet quality scores were calculated according to socio-demographic variables and t-tests were used to test for differences between groups. Data were analysed using Stata version 14 [34].

## Results

### Participant characteristics

NDNS dietary data were available for 2587 adolescents, 1282 boys and 1305 girls, aged 11–18 years. The mean age of the adolescents was 14.6 (SD 2.2) years and most participants (89%) were of white ethnicity. Area level deprivation measures show that the sample was highly representative of deprivation levels across the UK; 60% of participants lived in the three most deprived externally derived IMD quintiles. Individual markers of socio-economic status showed that 59% of participants came from households with income ≤£27,00 per year. Participant characteristics are shown in Table 1.

**Table 1:** Characteristics of adolescent participants (aged 11–18 years) from NDNS Years 1–8 of Rolling Programme

Characteristics	Boys	Girls	Total
n	1282	1305	2587
Age (years), mean (SD)	14.5 (2.3)	14.7 (2.2)	14.6 (2.2)
Ethnicity, n (%)			
White	1143 (89)	1166 (89)	2309 (89)
Non-white	138 (11)	138 (11)	276 (11)
IMD, n (%)			
Two least deprived quintiles	340 (41)	326 (39)	666 (40)
Three most deprived quintiles	493 (59)	506 (61)	999 (60)
Equivalised Household Income, n (%)			
≤£27,000	754 (59)	770 (59)	1524 (59)
>£27,000	393 (31)	404 (31)	797 (31)

### Principal component analysis

#### Aim 1: to identify a dietary pattern which describes diet quality in UK adolescents

The first component of the PCA results explained 3.0% of the variance within the dietary data. This component described a dietary pattern consistent with the UK dietary recommendations and represented a high-quality diet. High scores on this pattern were characterised by high consumption of fruit (apples, pears, other fruit), vegetables (tomatoes, leafy green vegetables, salad and raw vegetables and other vegetables), beans and pulses, wholegrains, nuts and seeds, and tap water, in addition to low consumption of sugar-sweetened carbonated beverages, chips, processed meats (manufactured coated chicken/turkey, burgers and kebabs and meat pies and pastries), white bread, crisps and savoury snacks, whole milk, baked beans and sugar added to tea, coffee, breakfast cereals etc.

### Diet quality scores

#### Aim 2: to create a reduced-item diet quality score

Two separate diet quality scores, one from the full 139-group and the other from the reduced 20-group, were created using the dietary pattern from the first component produced by the PCA. The coefficients for the 20 foods included in the 20-group diet quality score are shown in Table 2.

**Table 2:** PCA coefficients for the 20 indicator food groups included in the 20-group diet quality score

Food group	Principal Component Coefficient
*Positive Coefficients*	
Other fruit not canned	0.28
Nuts and seeds	0.24
Salad and other raw vegetables	0.23
Tap water only	0.21
Wholemeal bread	0.20
Apples and pears not canned	0.19
Other vegetables (including homemade dishes)	0.18
Beans and pulses (including ready meals and homemade dishes)	0.17
Leafy green vegetables not raw	0.16
Tomatoes (raw)	0.16
*Negative Coefficients*	
Sugar	−0.03
Baked beans	−0.04
Whole milk	−0.04
Crisps and savoury snacks	−0.05
White bread	−0.06
Meat pies and pastries (manufactured)	−0.06
Burgers and kebabs purchased	−0.10
Manufactured coated chicken/turkey products	−0.13
Chips, purchased including takeaway	−0.17
Soft drinks not low calorie, carbonated	−0.18

**Fig. 1:**
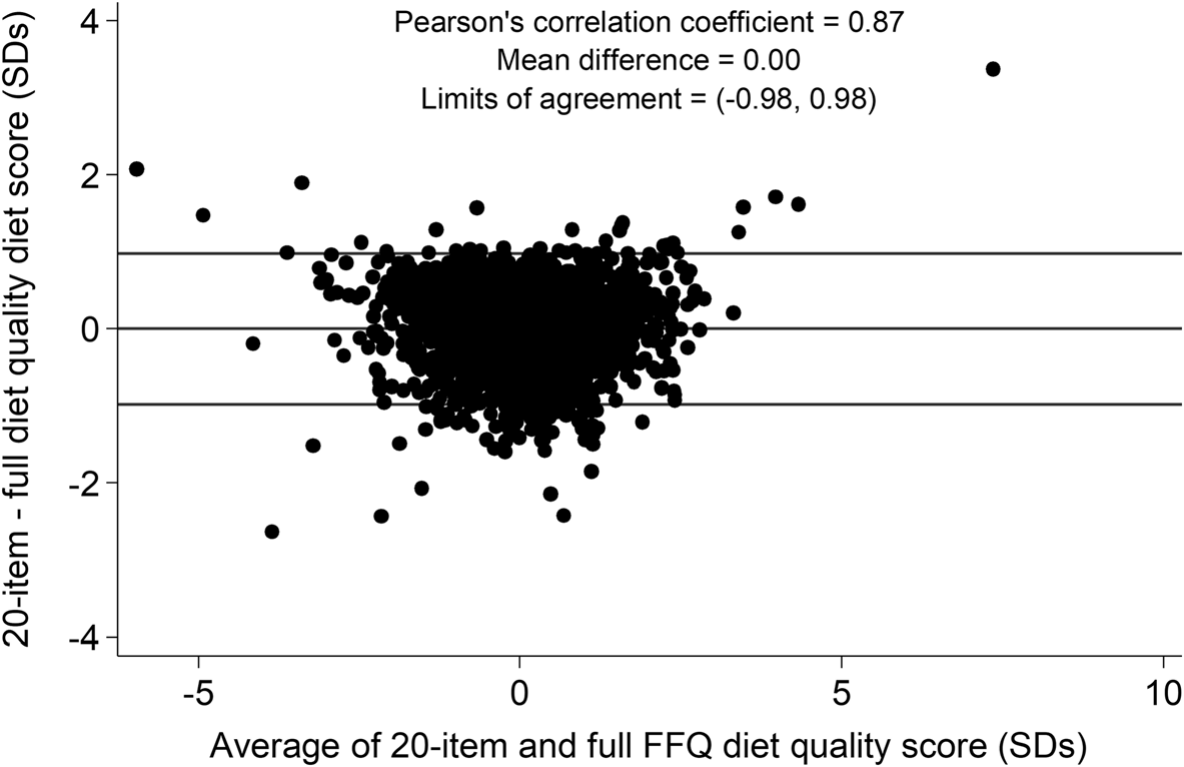
Bland-Altman plot for the agreement between the 139-group and 20-group diet quality scores

A correlation of 0.87 was observed between the full 139-group score and the reduced 20-group score. Figure 1 shows the agreement between the two scores. The mean difference between the scores was 0.00 SDs with Bland-Altman 95% limits of agreement of − 0.98 to 0.98 SDs.

The think-aloud qualitative interviews identified that the terminology used to describe the NDNS food groups was often not that used and understood by adolescents. A number of wording changes were made to the food group descriptions as a result of the think-aloud process. In addition to the changes in wording describing the food groups, example foods were provided for each food group. For example, “soft drinks not low calorie, carbonated” was changed to “fizzy drinks, not diet” and “other fruit not canned” was changed to “other fruit (not canned, not citrus, not apples and pears, and not bananas) e.g. grapes, plums, berries, mango, pineapple”. Additional file 3 shows the final version of the FFQ including changes to the wording which were identified through the think-aloud qualitative interviews.

### Associations with biomarkers

#### Aim 3: to investigate the associations between the diet quality score and objective biomarkers of nutritional status

Table 3 shows the correlation coefficients for both the full 139-group and reduced 20-group diet quality scores with the NDNS nutritional biomarkers.

**Table 3:** Spearman correlation coefficients for relationships between biomarkers and 139-group and 20-group diet quality scores

Biomarker	139-group Diet Quality Score			20-group Diet Quality Score		
	r_**S**_	***p***-value	n	r_**S**_	p-value	n
25-Hydroxy Vitamin D (nmol/L)	0.14	< 0.001	874	0.11	0.001	874
Holotranscobalamin (pmol/L)	0.22	0.004	171	0.12	0.10	171
Vitamin B12 (pmol/L)	0.21	< 0.001	872	0.15	< 0.001	872
Homocysteine (μmol/L)	−0.25	< 0.001	609	−0.18	< 0.001	609
Vitamin C (μmol/L)	0.30	< 0.001	845	0.27	< 0.001	845
Lutein (μmol/L)	0.24	< 0.001	862	0.23	< 0.001	862
Alpha-Cryptoxanthin (μmol/L)	0.23	< 0.001	862	0.20	< 0.001	862
Beta-Cryptoxanthin (μmol/L)	0.35	< 0.001	861	0.34	< 0.001	861
Lycopene (μmol/L)	0.10	0.002	861	0.09	0.006	861
Beta-Carotene (μmol/L)	0.24	< 0.001	861	0.18	< 0.001	861
Alpha-Carotene (μmol/L)	0.19	< 0.001	861	0.17	< 0.001	861
Total carotenoids (μmol/L)	0.25	< 0.001	861	0.21	< 0.001	861
Total serum folate (nmol/L)	0.42	< 0.001	177	0.39	< 0.001	177
Urinary sodium concentration (mmol/L)	−0.14	< 0.001	997	−0.14	< 0.001	997
Urinary potassium (mmol/L)	0.06	0.08	997	0.03	0.32	997

Positive correlations were observed between vitamin B12 and both the 139-group score (r_s_=0.21; *p*< 0.001) and the 20-group score (r_s_=0.15; p< 0.001). Similar trends were observed between holotranscobalamin and the 139-group score (r_s_=0.22; *p*=0.004). The correlation between holotranscobalamin and the 20-group score was in the expected direction, but weaker than the other correlations (r_s_=0.12; *p*=0.10). Total serum folate showed strong correlations with both the 139-group score (r_s_=0.42; *p*< 0.001) and the 20-group score (r_s_=0.39; p< 0.001). As expected, negative correlations were observed between homocysteine and both the 139-group score (r_s_= − 0.25; *p*< 0.001) and the 20-group diet quality score (r_s_=− 0.18; *p*< 0.001). 25-Hydroxy vitamin D showed a significant correlation in the expected direction with the 139-group and 20-group diet quality scores (r_s_=0.14; *p*=< 0.001 and r_s_ = 0.11, *P* = 0.001 respectively). Moderately positive correlations were observed between vitamin C and both the 139-group score (r_s_=0.30; p< 0.001) and 20-group diet quality score (r_s_=0.27; p< 0.001), and between total carotenoids and both the 139-group score (r_s_=0.25; p< 0.001) and 20-group diet quality score (r_s_=0.21; p< 0.001). Results in the expected direction were observed for the two included urinary biomarkers; i) negative correlations were observed between both diet quality scores and urinary sodium concentration (139-group: r_s_=− 0.14, p< 0.001; 20-group: r_s_=− 0.14, p< 0.001) and ii) weak but positive correlations were observed between both diet quality scores and urinary potassium (139-group: r_s_=0.06, *p*=0.08; 20-group: r_s_=0.03, *p*=0.32).

### Associations with socio-demographic characteristics

#### Aim 4: to investigate the associations between the diet quality score and socio-demographic characteristics

Table 4 shows the mean (SD) of the 20-group diet quality scores according to participant characteristics. Mean diet quality scores were lower among boys and white participants compared to girls and non-white participants respectively. A socio-economic gradient was also observed for diet quality scores with much lower scores among adolescents from the most deprived (IMD) areas and lower household income (≤£27,000) compared with those from the least deprived areas and higher income households (>£27,000) respectively.

**Table 4:** Mean (SD) 20-group diet quality score (SDs) by gender, ethnicity, income and IMD

Participant Characteristic	Mean (SD)	n	p-value
Gender			< 0.0001
Boys	−0.12 (0.97)	1282	
Girls	0.12 (1.01)	1305	
Ethnicity			< 0.0001
White	−0.04 (0.99)	2309	
Non-white	0.36 (0.98)	276	
IMD			< 0.0001
Two least deprived quintiles	0.18 (1.05)	666	
Three most deprived quintiles	−0.13 (0.96)	999	
Equivalised Household Income			< 0.0001
≤ £27,000	−0.14 (0.95)	1524	
> £27,000	0.26 (1.00)	797	

## Discussion

This study has shown that a short diet quality score, which assess the frequency of consumption of 20 food groups, can be used to assess overall diet quality in UK adolescents. PCA analysis using a national dietary dataset has identified a 139-group score, which represents the overall quality of diet in UK adolescents. From this, a 20-group dietary score was derived. Higher scores represent diets that adhere to UK dietary guidelines with greater intakes of fruits and vegetables and lower intakes of processed meats, and foods high in fat, salt and sugar. A strong correlation and adequate levels of agreement were observed between the full and short diet quality scores. Correlations in the expected directions between the full and short diet quality scores, and biomarkers of nutritional status were observed. The short 20-group diet quality score was also tested against known socio-demographic discriminators of diet quality and showed associations consistent with previous research [35].

In order to provide meaning to the magnitude of differences in diet quality that are described in this study, one possible interpretation is provided. As an illustrative example, the difference in diet quality score of 0.40SDs observed between adolescents from low and high income households using the 20-group score approximates to adolescents from high income households consuming on average an additional two portions of nuts and seeds and two fewer portions of chips per week compared with low income households.

The 20 indicator food groups identified in this study can be used to create an FFQ which can be used as a short and easy to implement tool eliminating the need for multiple days of dietary data collection. A limitation of previous literature, which has relied on the use of dietary indices to assess diet quality, is the requirement for multiple days-worth of dietary intake data usually collected via diet diaries or 24-h recalls [10, 11, 36–40]. The high levels of engagement and time required to accurately complete these assessments present difficulties when implementing them in large-scale studies, particularly with adolescents. The results from this study suggest that collecting data about the consumption of 20 key indicator food groups can assess UK adolescents’ compliance with an eating pattern that describes overall diet quality. This short FFQ has the potential to be used in various modes including traditional paper questions as well as in a digital format. Digital data collection reduces the researcher burden by removing the need for data entry. Even though there are limitations on the analyses that can be conducted using short tools, they remain useful as indicators of overall diet quality when individual nutrient and energy assessment are not the focus [7].

Diet quality in adolescents has most commonly been assessed using dietary indices. Data from the NDNS has previously been used to assess diet quality among UK adolescents using both the Diet Quality Index-Adolescents (DQI-A) [38] and the Mediterranean Diet Score [39]. In the US, the Healthy Eating Index (HEI) has been used to assess adolescent diet quality [36, 37]. A number of adolescent-specific scores have also been developed to assess adolescents’ compliance with healthy eating guidelines in the US [10], Europe [11] and New Zealand [40]. All of these indices reflect the main principles of healthy eating guidelines common across countries. These include high consumption of fruit, vegetable, wholegrain products and lean meat, although with some differences according to the specific recommendations in the national guidelines. Two of the scores assess the consumption of non-recommended foods such a sugar sweetened beverages and snack foods as these are frequently consumed by adolescent populations [10, 11]. In addition to considering the recommendations from healthy eating guidelines, the New Zealand DQI-A score and the DQI-A score also include diet diversity, with higher scores being given if a greater variety of foods are consumed [11, 40]. The dietary scores developed in our study use a different approach. While still reflecting the UK Healthy Eating Guidelines they do not, unlike diet quality indices, use the recommendations to inform the calculation of the diet quality score. As many UK adolescents are failing to meet Public Health England’s recommendations for dietary intake, using a data-driven approach as we have in this study, allows researchers to base diet quality scores on what people are actually eating [1, 2]. Such approaches may be particularly helpful for intervention studies because they can identify important food groups to target as part of healthy eating interventions.

This study is not the first to use a data-driven approach to assess dietary patterns among adolescents. Data from the UK Avon Longitudinal Study of Parents and Children (ALSPAC) study identified a “traditional/health conscious” pattern as the strongest among 13 year olds from dual-source parent-child FFQs [41]. Even though the food groups used within the ALSPAC FFQ were different to the NDNS food groups, many of the foods contributing most to the “traditional/health conscious” pattern were similar to the strongest variables identified in this study. However, a number of food groups such as fish, eggs, rice, pasta and potatoes were also strong positive contributors to the ALSPAC pattern but were not replicated in the 20-group diet quality score in our study. One possible reason for these differences is that the ALSPAC dietary data were collected from participants aged 13 years. It is likely that dietary patterns change during adolescence. The age range used in the NDNS is much wider (11–18 years) and thus captures the possible differences in consumption over this age range.

This study showed clear sociodemographic differences in adolescent diet quality scores. Higher diet quality scores were observed in girls compared to boys. This finding is consistent with previous research in UK and Australian adolescents [41, 42]. Socio-economic differences in diet quality score were also apparent, with adolescents from households in more deprived neighbourhoods and those from households with incomes below £27,000 per annum having lower diet quality. In previous research among French and US populations, adolescents of lower socio-economic status have been shown to have higher consumption of energy-dense, nutritionally poor foods and lower consumption of fruits and vegetables [43, 44]. The consistency of these results with the demographic associations we identified provide confidence in the robustness of the diet quality score identified in our study. Nevertheless, further work would be needed to determine if this diet quality score is suitable for use with adolescents from countries outside the UK.

Our study is one of few to compare adolescent diet quality with nutritional biomarkers and found associations in the expected direction between diet quality scores and all biomarkers of nutritional status assessed. Positive associations were observed with biomarkers that have previously been associated with healthier dietary patterns in adult populations such as 25-hydroxy vitamin D [24], total carotenoids [25, 26], total serum folate [13] and vitamin C [15, 29]. Healthy nutrition scores among German adolescents have been shown to be inversely associated with homocysteine and positively associated with serum folate [45]. In addition, Vyncke and colleagues showed positive associations between the DQI-A score and biomarkers reflecting long-term dietary intake, 25-hydroxy vitamin D and holotranscobalamin, but weaker associations with other plasma biomarkers which represent short to medium term dietary intake, suggesting that the DQI-A score may only be a valid assessment measure of long term dietary [11]. Comparing our results with these findings indicates that the short 20-group FFQ is a useful tool to assess diet quality and provides an adequate proxy for nutritional status. The majority of correlations observed in this study were significant but not always large in magnitude. Each individual biomarker reflects one element of nutritional status and as a result, extremely strong correlations would not be expected when comparing biomarkers with dietary patterns which provide a more rounded view of dietary intake. It is however worth noting that the correlations observed in this study are similar in magnitude to results from others studies when healthy dietary patterns were compared to: serum folate (r(s)= 0.31) [13]; total carotenoids (β 0.32 (95%CI 0.26, 0.38) [30]; 25-Hydroxy vitamin D (β 0·301 (95%CI 0·164, 0·438) [11]; and homocysteine (r= − 0·068) [45].

### Strength and limitations

A strength of our study was the use of nationally representative data from the NDNS. This national dataset gives confidence that the 20-group diet quality score is representative of and suitable for use with, UK adolescents. Self-report dietary assessment methods have been shown to be prone to under-reporting, resulting in inaccurate assessment of nutrient and energy intake [7]. However, in line with recommendations, this study has compared the diet quality score with objective measures of nutritional status [7]. Some study limitations must also be acknowledged. Wide variation in dietary intakes has been shown between countries, so the items considered in this score may not be the best indicators of a healthy dietary pattern in adolescent populations from other countries [46]. This study did not test the diet quality score against an already established, validated dietary assessment method. Future research could complete such a comparison to confirm the validity of the diet quality score and strengthen confidence in the ability of the tool to accurately assess the quality of diet in UK adolescents. Another limitation is that the strongest pattern identified through the PCA, which represents a high-quality diet, explained only 3% of variation within the data. This finding may be due to the large number of variables included in the PCA analysis [30]. Previous research has identified that as the number and specificity of dietary variables included in a PCA increases, the amount of variation explained by each factor decreases [47]. This study aimed to identify the key indicator foods that describe a high-quality diet in UK adolescents. Using a large number of specific food categories may therefore provide a greater level of granularity about the indicator foods of better and poorer adolescent dietary quality, and that is likely to be a more important consideration than the overall level of variation described by each PCA component.

Additionally, data from the NDNS food-level dataset uses a pre-defined categorical system to group food products whereby some foods within the same category may vary to a greater extent in their nutritional composition than other food grouping systems. These food groups also present difficulties when developing a FFQ because some groups, for example ‘other fruit’, are difficult to ask as standalone FFQ questions. The face validity work, conducted as part of this study, helped to overcome this limitation and ensured that the wording of this short FFQ is understandable and meaningful to adolescents.

## Conclusions

The results from this study show that a short 20-item FFQ can be used to assess overall diet quality of UK adolescents. Short FFQs have clear advantages for use in large research studies, where time and resources are limited, as they are simple and brief to administer. Such self-report tools may be particularly useful when research requires an overall picture of diet quality and/or ranking of participants rather than details of absolute nutrient or energy intake. The associations observed with nutritional biomarkers and known socio-demographic discriminators of diet quality provide confidence in the robustness of the 20-item score to assess diet quality in UK adolescents.

## Supplementary Information

**Additional file 1.** STROBE-nut checklist.

**Additional file 2.** How to create the short FFQ diet quality score for adolescents.

**Additional file 3.** Short Food Frequency Questionnaire.

## Data Availability

The datasets supporting the conclusions of this article are available in the UK Data Service archives, [Unique persistent identifier: 10.5255/UKDA-SN-6533-15; Hyperlink to datasets: https://beta.ukdataservice.ac.uk/datacatalogue/studies/study?id=6533].

## Abbreviations

FFQ: Food Frequency Questionnaire
UK: United Kingdom
PCA: Principal Component Analysis
NDNS: National Diet and Nutrition Survey
DQI-A: Diet Quality Index- Adolescents
IMD: Index of Multiple Deprivation
IQR: Inter Quartile Range
SD: Standard Deviation
NZDQI-A: New Zealand Diet Quality Index- Adolescents
