# Supplementary material for: Development of a short food frequency questionnaire to assess diet quality in UK adolescents using the National Diet and Nutrition Survey
Source: Nutr J. 2021 Jan 12;20:5. doi: 10.1186/s12937-020-00658-1 (PMC7802176; doi:10.1186/s12937-020-00658-1)
Supplement: Supplementary file 2 — Additional file 2. How to create the short FFQ diet quality score for adolescents. [file 12937_2020_658_MOESM2_ESM.docx]

**Appendix B**

**How to create the short FFQ diet quality score for adolescents**

These values are based on analyses performed amongst adolescents in the NDNS data.

| **Food** | **Mean** | **SD** | **Coefficient** | **Units** |
| --- | --- | --- | --- | --- |
| Other fruit not canned (40R) | 1.817195 | 3.697939 | 0.2752 | Frequency per week |
| Nuts and seeds (56R) | 0.347249 | 1.994155 | 0.2358 | Frequency per week |
| Salad and other raw vegetables (36B) | 2.674494 | 4.104228 | 0.2251 | Frequency per week |
| Tap water only (51R) | 6.252062 | 8.199518 | 0.2120 | Frequency per week |
| Wholemeal bread (3R) | 0.901946 | 2.36824 | 0.2026 | Frequency per week |
| Apples and pears not canned (40A) | 1.289557 | 2.19712 | 0.1855 | Frequency per week |
| Other vegetables (including homemade dishes) (37M) | 2.482831 | 5.765391 | 0.1750 | Frequency per week |
| Beans and pulses (including ready meals and homemade dishes) (37I) | 0.522001 | 3.764656 | 0.1748 | Frequency per week |
| Leafy green vegetables not raw (37D) | 0.629784 | 1.207219 | 0.1639 | Frequency per week |
| Tomatoes (raw) (36C) | 0.679165 | 1.408425 | 0.1601 | Frequency per week |
| Sugar (41A) | 2.790394 | 4.583202 | -0.0314 | Frequency per week |
| Baked beans (37C) | 0.720204 | 1.298639 | -0.0365 | Frequency per week |
| Whole milk (10R) | 1.598699 | 4.124837 | -0.0446 | Frequency per week |
| Crisps and savoury snacks (42R) | 2.984989 | 3.067756 | -0.0541 | Frequency per week |
| White bread (not high fibre or multiseed) (2R) | 5.698492 | 4.193377 | -0.0568 | Frequency per week |
| Meat pies and pastries (manufactured) (31A) | 0.743203 | 2.306639 | -0.0638 | Frequency per week |
| Burgers and kebabs purchased (29R) | 0.547481 | 1.399524 | -0.1011 | Frequency per week |
| Manufactured coated chicken/turkey products (26A) | 0.989434 | 1.773349 | -0.1280 | Frequency per week |
| Chips, purchased including takeaway (38A) | 1.577954 | 1.777083 | -0.1703 | Frequency per week |
| Soft drinks not low calorie carbonated (57B) | 3.303827 | 4.274825 | -0.1845 | Frequency per week |

**Steps to derive diet quality score**

- For each variable in a new dataset, ensure the data are in the units described in the table.
- Check that the means and SDs of each variable are (very) approximately the same as those in the table. If they are not this may indicate a problem with the units.
- Create ‘standardised’ food variables by subtracting the means and dividing by the SDs in the above table.
- Create the diet quality score by multiplying the coefficient for each food by the standardised food variables, and summing them, resulting in one score for each subject.
- Add 5 on to these scores and then take the natural log of them.
- This score has no units. Subtract the mean and divide by the SD of the score in the dataset to get a score with internal standard deviation units. Alternatively subtract 1.587165 and divide by 0.2062927 to get a score in SDs of the original dataset.

**CAUTION**

This short FFQ assesses a particular axis of variation in diet (diet quality) but is not valid to study other aspects of diet.
