# Supplementary material for: Development of a short food frequency questionnaire to assess diet quality in UK adolescents using the National Diet and Nutrition Survey
Source: Nutr J. 2021 Jan 12;20:5. doi: 10.1186/s12937-020-00658-1 (PMC7802176; doi:10.1186/s12937-020-00658-1)
Supplement: Supplementary file 3 — Additional file 3. Short Food Frequency Questionnaire. [file 12937_2020_658_MOESM3_ESM.docx]

**Appendix C**

| Short Food Frequency Questionnaire Adolescents aged 11-18 years |
| --- |

In this section we would like to know about some of the foods you eat and how often you eat them. Please tick one box in each row.

Think about the foods you have eaten in the past **MONTH**

| **Over the past month how many times have you eaten…** | **Never** | **Once a Month** | **Once every two weeks** | **1-2 Times per Week** | **3-6 Times per Week** | **Once a day** | **More than once a day** |
| --- | --- | --- | --- | --- | --- | --- | --- |
| **Apples and Pears not canned** [Including raw, baked, stewed, dried] |  |  |  |  |  |  |  |
| **Other fruit (Not canned, not citrus, not apples and pears, and not bananas)** e.g. grapes, plums, berries, mango, pineapple |  |  |  |  |  |  |  |
| **Nuts and Seeds** e.g. cashew nuts, coconut, salted peanuts, nut butters, tahini, pumpkin seeds |  |  |  |  |  |  |  |
| **Crisps and savoury snacks** (Include all potato based snacks and grain based snacks e.g. pretzels, popcorn (not sweet), tortilla chips) |  |  |  |  |  |  |  |
| **Salad and other raw vegetables** (Including all types of raw vegetables. Purchased or homemade) e.g. side salad, coleslaw, guacamole |  |  |  |  |  |  |  |
| **Beans, lentils and chickpeas** (**Not baked beans)**  (Include dishes based on lentils, dried beans and pulses)  e.g. kidney beans, black beans, hummus, chickpea curry, bean stew |  |  |  |  |  |  |  |
| **Tomatoes** (fresh, not cooked, not tinned) |  |  |  |  |  |  |  |
| **Leafy green vegetables** (Include cooked and canned) e.g. spinach, kale, broccoli, cabbage, Brussels sprouts |  |  |  |  |  |  |  |
| **Baked beans** (Include canned baked beans in sauce. Including baked beans with additions e.g. sausages) |  |  |  |  |  |  |  |
| **Other vegetable dishes** (Cooked vegetable dishes usually forming a main meal)  (Vegetable curries, casseroles and stews, vegetable pies, vegetable lasagne, vegetable stir-fry) |  |  |  |  |  |  |  |
| **Meat pies and pastries** (Including sausage rolls, pasties, meat samosas) |  |  |  |  |  |  |  |
| **Burgers and kebabs** (Purchased or takeaway burgers or kebabs) |  |  |  |  |  |  |  |
| **Breaded or battered chicken/ turkey products** (Including fried chicken, nuggets, chicken kievs, chicken burgers) |  |  |  |  |  |  |  |
| **Chips** (Include any types of purchased or takeaway chips e.g. frozen, oven, microwave, purchased from takeaway or restaurant) |  |  |  |  |  |  |  |
| **All types of white bread** (Including French stick, white pitta bread, English muffins, plain bagels, white tortillas, white rolls) |  |  |  |  |  |  |  |
| **All types of 100% Wholemeal bread** (Not granary) (Including wholemeal pitta bread, wholemeal bagels, wholemeal tortillas, wholemeal rolls) |  |  |  |  |  |  |  |
| **Whole "Blue-top" Milk** (Whole cow's milk. Do not include any other types of milk) |  |  |  |  |  |  |  |
| **Sugar** (Added by you to cereals, tea and coffee, and desserts)  (Include sugar, golden syrup and maple syrup) |  |  |  |  |  |  |  |
| **Tap water** (Not including water drank with squash) |  |  |  |  |  |  |  |
| **Fizzy drinks and Energy drinks** (Not diet or low calorie) |  |  |  |  |  |  |  |
